# Guangdong Biobank Cohort (GDBC) study

**DOI:** 10.1007/s10654-025-01320-y

**Published:** 2026-01-13

**Authors:** Yong-Qiao He, Wen-Qiong Xue, Hua Diao, Ji-Yun Zhan, Ming-Fang Ji, Da-Wei Yang, Yi Zhao, Chang-Mi Deng, Zi-Yi Wu, Ting Zhou, Ying Liao, Mei-Qi Zheng, Wen-Li Zhang, Yi-Jing Jia, Lei-Lei Yuan, Lu-Ting Luo, Dan-Hua Li, Tong-Min Wang, Xia-Ting Tong, Yan Du, Ling-Ling Tang, Jing-Wen Huang, Chang-ling Huang, Zhi-Yang Zhao, Yan-Xia Wu, Lian-Jing Cao, Si-Qi Dong, Fang Wang, Cheng-Tao Jiang, Ruo-Wen Xiao, Wen-Bin Zhang, Xue-Yin Chen, Qiao-Ling Wang, Qiao-Yun Liu, Yue-Ze Zhao, Cao-Li Tang, Lin Ma, Xiao-Hui Zheng, Pei-Fen Zhang, Xi-Zhao Li, Shao-Dan Zhang, Ye-Zhu Hu, Xia Yu, Biao-Hua Wu, Fu-Gui Li, Jian-Hua Wu, Bi-Sen Deng, Xue-Jun Liang, Wei-Hua Jia

**Affiliations:** 1 State Key Laboratory of Oncology in South China, Guangdong Provincial Clinical Research Center for Cancer, Guangdong Key Laboratory of Nasopharyngeal Carcinoma Diagnosis and Therapy, Sun Yat-sen University Cancer Center, 651 Dong Feng East Road, Guangzhou, Guangdong P. R. China; 2 School of Public Health, Sun Yat-Sen University, Guangzhou, Guangdong P. R. China; 3 Department of Gastroenterology, Third Military Medical University Xinqiao Hospital, Chongqing, P.R. China; 4 Public Health Service Center of Xiaolan Town, Zhongshan, Guangdong P. R. China; 5 Cancer Research Institute of Zhongshan City, Zhongshan City People’s Hospital, Zhongshan, Guangdong P. R. China; 6 Guangdong Provincial Center for Disease Control and Prevention, Guangzhou, Guangdong P. R. China; 7 Guangzhou Women and Children’s Medical Center, Guangzhou Medical University, Guangzhou, Guangdong P. R. China; 8 Clinical Oncology School of Fujian Medical University, Fujian Cancer Hospital, Fuzhou, Fujian, P. R. China; 9 Shenzhen Maternity and Child Healthcare Hospital, Southern Medical University, Shenzhen, Guangdong P. R. China; 10 Shenzhen Futian Center for Chronic Disease Control, Shenzhen, Guangdong P. R. China; 11 Foshan Key Laboratory of Precision Therapy in Oncology and Neurology, Department of Pulmonary Oncology, The First People’s Hospital of Foshan, Foshan, Guangdong P. R. China; 12 Zengcheng District Center for Disease Control and Prevention, Guangzhou, Guangdong P. R. China; 13 Gastrointestinal Cancer Institute/Pancreatic Disease Institute, The Affiliated Hospital of Qingdao University, Qingdao, Shandong, P. R. China; 14 Department of Radiation Oncology, Affiliated Cancer Hospital & Institute of Guangzhou Medical University, Guangzhou, Guangdong P. R. China; 15 Department of Oncology, The First Hospital of Lanzhou University, Lanzhou, Gansu, P.R. China; 16 Department of Urology, The First Affiliated Hospital of Guangzhou Medical University, Guangzhou, Guangdong P. R. China; 17 The Eighth Affiliated Hospital of Sun Yat-sen University, Shenzhen, Guangdong P. R. China

**Keywords:** Population-based cohort, Cloud-based multidimensional database, Gene–environment interactions, Oral microbiota, Non-communicable disease, Longitudinal study

## Abstract

The global rise of non-communicable diseases (NCDs) presents an urgent public health challenge, particularly in regions undergoing rapid economic and demographic transitions. Guangdong Province, China’s most populous and economically advanced region, is experiencing a substantial and accelerating burden of NCDs. However, large-scale, population-based cohorts from this region remain scarce, limiting insights into region-specific disease determinants and prevention strategies. The Guangdong Biobank Cohort (GDBC) was established in 2017, enrolling 35,081 participants aged 40–84 years from urban and rural areas of Zhongshan City in the Pearl River Delta. At baseline, comprehensive data on 346 variables—including lifestyle, environmental exposures, medical histories, physical examinations, and laboratory profiles—were collected via a cloud-based member management information system (MMIS), alongside blood and saliva samples for biobanking. A sub-cohort underwent genome-wide genotyping (*N* = 2,530) and oral microbiome profiling via 16 S rRNA sequencing (*N* = 2,049). During dynamic follow-up, 44.2% (*N* = 15,499) completed Phase I resurvey with repeated measurements and updated biospecimens. Disease outcomes, including hypertension, diabetes, and cancer, were ascertained through active surveillance and regional registry linkage until December 2023. Baseline prevalence of hypertension, diabetes, and cancer was 25.3%, 8.0%, and 3.6%, respectively. Over follow-up, 1,767 hypertension cases, 814 diabetes cases, and 558 cancers were recorded, yielding crude incidence rates of 1,804.6, 649.7, and 423.1 per 100,000 person-years, respectively. The GDBC provides a comprehensive, dynamically updated resource to dissect gene–microbiome–environment interactions and develop precision prevention strategies to inform public health policies.

**Supplementary Information:**

The online version contains supplementary material available at 10.1007/s10654-025-01320-y.

## Why was the cohort set up?

Non-communicable diseases (NCDs), primarily cardiovascular diseases (CVD) and cancer, account for 74% of global mortality, causing over 41 million deaths annually [1, 2]. In China, this burden is even more pronounced, with NCDs responsible for 88.5% of total deaths, of which CVD and cancer contribute 47.1% and 24.1%, respectively [3–5]. This escalating crisis is driven by population aging, rapid economic development, and profound changes in environmental exposures and lifestyles. Guangdong Province, as one of China’s most economically developed regions with a population of 126 million [6], exemplifies this growing NCD crisis [7, 8]. However, Guangdong has been underrepresented in major national cohort studies. For instance, the China Kadoorie Biobank (CKB) [9, 10], a cornerstone of NCD research, recruited 500,000 participants but excluded Guangdong entirely, leaving critical gaps in understanding region-specific disease determinants.

The NCDs risks in Guangdong arise from a complex interplay of environmental, genetic, infectious, and lifestyle factors, yet the interactions among these determinants remain poorly understood due to a lack of large, population-based studies in this region [11, 12]. Guangdong’s rapid industrialization has resulted in high exposure to airborne pollutants (PM2.5, NO2) and occupational carcinogens (e.g., benzene), which have been linked to increased risks of cancer, cardiovascular diseases, and metabolic disorders [13–15]. The subtropical climate, with year-round high humidity, fosters indoor mold proliferation, which has been associated with respiratory diseases and allergic conditions [16, 17]. Additionally, lifestyle and behavioral factors further contribute to NCD risks, including a shift toward sedentary occupations [18] and dietary habits such as frequent consumption of seafood, preserved foods (e.g., salted fish, a Group 1 carcinogen linked to nasopharyngeal carcinoma (NPC) [19, 20], incense burning [21, 22], and traditional Cantonese herbal teas [23]. A particularly striking feature of Guangdong’s disease profile is its exceptionally high NPC incidence, which is 20- to 50-fold higher than in low-risk regions [24–26]. This elevated risk can be partly explained by the universal Epstein-Barr virus (EBV) seroprevalence, which interacts with host genetic variants, particularly those in the HLA region, leading to heightened NPC susceptibility [27, 28]. Despite these well-documented risk factors, the cumulative and synergistic effects of genetic, infectious, and environmental exposures on NCD risk remain largely unexplored.

Therefore, the Guangdong Biobank Cohort (GDBC, 2017–present, *N* = 35,081) was established under support of the China’s National Key R&D Program (No. 2016YFC1302700). This cohort integrates an electronic data collection system, leveraging electronic questionnaires and a cloud-based information platform to enhance data accuracy, follow-up efficiency, and participant engagement. By incorporating multi-omics approaches (genomics, oral microbiome, serological biomarkers), comprehensive environmental assessments, and long-term follow-up data, GDBC provides an opportunity to systematically evaluate the interactions between genetic susceptibility, environmental exposures, infectious agents, and lifestyle factors. The findings from this cohort will contribute to precision prevention and risk stratification, facilitate the refinement of cancer and chronic disease screening programs, identify novel biomarkers, and inform targeted prevention strategies for high-risk populations in Southern China and beyond.

## Who is in the cohort?

### Cohort design and setting

The Guangdong Biobank Cohort is an ongoing population-based prospective cohort initiated in 2016 in Zhongshan City, Guangdong Province, a key commercial and healthcare hub in the central Pearl River Delta (PRD) and an integral part of the Guangdong-Hong Kong-Macao Greater Bay Area. Xiaolan town, a rapidly urbanizing region within Zhongshan, was selected for its well-developed healthcare infrastructure, stable resident population, and high-quality disease surveillance system, which ensure long-term follow-up feasibility and high data reliability. With a registered permanent population of 288,600 (2023) and a GDP per capita of ¥87,000 (USD 12,200), Xiaolan provides an optimal setting for studying urbanization-related health transitions and non-communicable diseases (NCDs) epidemiology.

The town has 16 community health service centers, offering standardized health examinations, chronic disease management, and cancer screening programs, ensuring universal healthcare coverage and high participant retention [29]. Since the 1980 s, Zhongshan has operated one of China’s earliest population-based cancer registries, incorporated into the International Agency for Research on Cancer (IARC)’s Cancer Incidence in Five Continents (CI5) database. Additionally, Zhongshan has pioneered systematic screening programs for NPC and liver cancer [30], both of which have elevated incidence rates in Southern China. The cohort is supported by a unified electronic health record system, established in 2010, which enables seamless baseline data capture and longitudinal tracking. This system integrates data from annual health check-ups, outpatient visits, and hospitalization records, with NCD registries (such as hypertension and diabetes) to achieve high follow-up compliance.

### Participant recruitment and inclusion criteria

Participants were recruited from all 16 community health service centers across Xiaolan. Recruitment quotas and age distribution targets were allocated to each community healthcare service center based on the population size of their service area. The inclusion criteria were as follows: (1) Registered permanent residents of Zhongshan, Guangdong, confirmed by the household registration (hukou) system; (2) Residence in Guangdong for at least 10 years prior to enrollment; (3) Aged 40 to 84 years at enrollment; (4) Physically capable and fully conscious to complete the baseline survey and physical examination; (5) Provided written informed consent after fully understanding the study objectives.

During the initial enrollment phase, community health service centers in this study recruited 38,301 residents who expressed willingness to participate. Following sequential registration and eligibility screening, 3,093 individuals were excluded: 3,005 were outside the target age range (40–84 years), and 88 declined participation after initial registration (questionnaire, physical examination, and laboratory tests). During follow-up, an additional 127 participants were excluded because their records were unavailable across both active and passive follow-up systems.

## What has been measured?

### Baseline survey

**Fig. 1:**
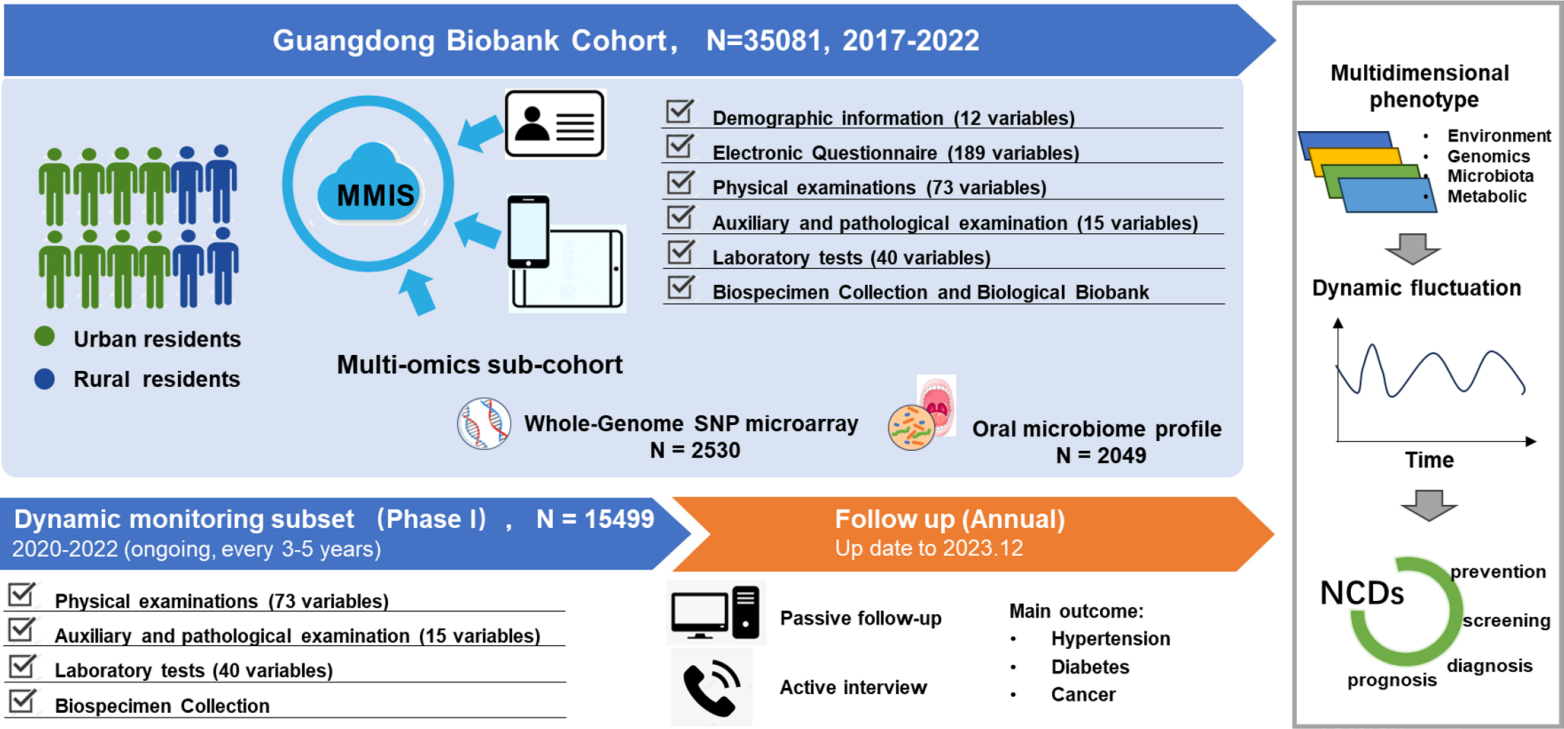
Baseline recruitment, data collection, biobanking, and dynamic follow-up structure of the Guangdong Biobank Cohort (GDBC)

The baseline assessments were conducted from October 2017 to February 2022, with longitudinal follow-ups planned every 3–5 years. To ensure efficient participant management, we developed a Member Management Information System (MMIS), which integrates electronic questionnaires and participant records on a secure commercial cloud server (Alibaba Cloud). Eligible residents were invited to their nearest community healthcare service center for physical examinations. Upon arrival, participants were registered using their unique identification card number, followed by a face-to-face interview and physical examination conducted by trained healthcare personnel (Fig. 1).

The GDBC cohort enrolled 35,081 adults, covering approximately 30–40% of local residents aged 40–84 years (based on 2016 Xiaolan Town Public Health Service Center data, Table S1, Fig. S1).

### Electronic questionnaire investigation

Participants completed structured face-to-face interviews using the electronic questionnaire integrated into MMIS, administered by trained healthcare personnel via tablet computers or mobile devices. The questionnaire was designed with reference to the China Kadoorie Biobank to ensure comprehensive coverage of key epidemiological variables while adapting to the unique regional characteristics of Guangdong. It encompassed 205 items across multiple domains (Table 1). Demographic information included age, gender, education level, marital status, nationality, birth data, family address, and rurality. Lifestyle factors assessed cigarette smoking, alcohol consumption, physical activity, dietary habits, and Guangdong-specific lifestyle behaviors. Oral hygiene was evaluated through indicators such as frequency of teeth brushing, age started teeth brushing, persistent time of teeth brushing, loss of tooth, number of dental caries, age of first tooth loss, and toothache symptoms. Personal medical history documented the participants’ history of NCDs such as hypertension, diabetes, cancers, as well as results of previous endoscopies, CT scan, X-ray, EBV antibody testing, HPV, H pylori testing and other related examinations, along with medication history recording the use of antibiotics, calcium supplements, fish oil, vitamins, metformin, NSAIDs, and other commonly used medications. Family disease history focused on common cancers, including lung, breast, and colorectal cancer, as well as other chronic diseases among parents, siblings, and children. For female participants, reproductive history was also collected, covering age at menarche, age at menopause, menstrual cycle characteristics, pregnancy history, hormone therapy use, age at first birth, and breastfeeding history.

**Table 1:** Baseline data collection overview in the Guangdong biobank cohort study

Measurement	No. of variables	Variables
Demographic characteristics	12	Age when recruited, sex, nationality, education, marital status, medical insurance type, family address, birth date, residence area (urban/rural), community health center when recruited.
Lifestyle factors	69	
Dietary factors	16	Consumption and/or frequency of cereals, vegetables, fruits, white meat, red meat, sausages, salted fish, pickled vegetables, slow-cooked soup, soy products and milk, dietary habit.
Smoking	8	Smoking status, age started smoking, cigarettes smoked per day, duration of smoking, smoking cessation, frequency and duration of passive smoking
Alcohol drinking	10	Alcohol drinking status, age started drinking, duration of drinking, alcohol type, consumption of alcohol, drinking cessation, drunkenness
Tea drinking	4	Tea drinking status, duration, type of tea consumed
Incense burning	2	Incense burning status, frequency and location of incense burning
Exercise and activity	16	Type of Exercise, frequency of high-level and middle to low-level exercise, persistent period of exercise habit, duration of exercise, time of housework per week, time of sedentary work per day, type of work, work patterns (primarily sitting or standing)
Occupational exposure	3	Type of occupational exposure, duration of exposure, work type
Oral hygiene	10	Frequency of tooth brushing, age started tooth brushing, persistent time of tooth brushing, loss of tooth, number of dental caries, age of first tooth loss, toothache
Personal medical history	69	Personal history of hypertension, diabetes, coronary heart disease, cancer, tuberculosis, mental disorder, inflammatory bowel disease and relevant symptoms, history and results of previous laboratory tests and auxiliary examinations for NCD screening, medication history
Family history	38	Birth weight, number of siblings, family history of cancer and other chronic diseases
Menstrual and reproductive history	17	Age of menarche, age of menopause, menstrual period, menstrual regularity, number of births, menstrual blood loss, age at first childbirth, history of breastfeeding, number of spontaneous abortions, number of induced abortion (women only), history of oral contraceptive use
Physical examination	73	Height, weight, waist circumference, hip circumference, body mass index, grip strength, bone mass, heart rate, breath count, blood pressure in both left and right arm, the percentages of body fat, muscle, bone mass, water, visceral fat, subcutaneous fat, skeletal muscle, basal metabolic rate, and physical examination of the oral cavity, skin, and heart, vision examination and abdominal physical examination
Laboratory test	39	
Routine blood test	11	Hemoglobin, white blood cell count, red blood cell count, platelet count, lymphocyte count, lymphocyte percentage, mean corpuscular volume, mean corpuscular hemoglobin, mean corpuscular hemoglobin concentration, mean platelet volume, platelet distribution width
Routine urine test	6	Urine glucose, urine ketones, urine protein, urine occult blood, other Abnormalities, summary of routine urine test
Lipid test	5	Total cholesterol, triglycerides, low-density lipoprotein (LDL) cholesterol, high-density lipoprotein (HDL) cholesterol, summary of lipid test
Blood glucose test	3	Fasting blood glucose (mmol/L), glycated hemoglobin (HbA1c) test, summary of blood glucose test
Hepatic function	5	Alanine aminotransferase (ALT), aspartate aminotransferase (AST), alanine to aspartate ratio (ALT/AST ratio), total bilirubin, summary of hepatic function test
Renal function	4	Serum creatinine, blood urea nitrogen, uric acid, summary of renal function test
Tumor marker	5	HBsAg, fecal occult blood test, alpha-fetoprotein (AFP), carcinoembryonic antigen (CEA), EBV VCA-IgA
Electrocardiogram	3	Summary of the electrocardiogram, abnormalities, explanation
Ultrasound examination	8	Liver, gallbladder, pancreas, spleen, kidney, breast, summary of ultrasound examinations and abnormalities
X-ray test	2	Abnormalities (Yes/No), explanation
Pap test (women only)	2	Abnormalities (Yes/No), explanation
Cognitive and emotional assessments	5	Assessment of Intelligence, cognitive function, emotional state, depression and self-care ability
Comprehensive health assessments	4	Self-reported health assessment, physician advice, health recommendations
Health advice related	5	Lifestyle counseling, target setting for health goal, such as lose weight, vaccinations and other health advice.

Each interview lasted approximately 30–40 min, and the system was equipped with a real-time audio recording feature. To ensure data quality, approximately 2–5% of interviews were randomly selected for quality control, where designated personnel reviewed the recordings and cross-checked responses for accuracy and consistency. All audio recordings were permanently stored on a cloud-based server, ensuring data traceability and reliability.

### Physical examination

A comprehensive physical examination was conducted for all participants during the baseline survey, following standardized protocols. Anthropometric measurements included height, weight, waist circumference, hip circumference, and grip strength. Height and weight were measured for each participant using an integrated digital height-weight scale (DHM-300G, Zhengzhou Dingheng Electronics Technology Co., Ltd.). Participants were required to remove shoes and wear light clothing. Height was measured with participants standing upright, heels and knees together, using a stadiometer, while weight was recorded using a calibrated digital scale. Waist circumference was measured at the midpoint between the superior border of the iliac crest and the lower rib margin, with a flexible measuring tape placed horizontally around the abdomen. Hip circumference was recorded at the widest part of the buttocks, ensuring the tape remained parallel to the floor. Grip strength was assessed using a hand dynamometer (Xiangshan dynamometer, Manufacturer: Guangdong Xiangshan Weighing Instrument Group Co., Ltd.), with each participant performing two trials for both hands, and the average value recorded. Body composition analysis was performed using a bioelectrical impedance analyzer (PICOOC, Manufacturer: Youpin international technology [Shenzhen] co., ltd), which measured the percentages of body fat, bone and muscle mass, visceral fat, water, skeletal muscle, subcutaneous fat, and basal metabolic rate. Vital sign assessments included blood pressure, heart rate, and respiratory rate. Blood pressure was measured in both upper arms, aligned with heart level, using an automated blood pressure monitor (Yuwell, Manufacturer: Jiangsu Yuyue Medical Equipment & Supply Co., Ltd.) after participants had rested for at least 10 min. If the initial test was abnormal, a repeat measurement was conducted after at least 30 min. Heart rate was obtained from the blood pressure monitor, while respiratory rate was manually counted using a stopwatch.

In addition, head and neck examinations were conducted to assess thyroid abnormalities, lymph node enlargement, and other structural anomalies. Vision assessment included visual acuity testing and screening for refractive errors. Dental examinations evaluated oral hygiene, periodontal disease, cavities, missing teeth, and other dental conditions. Gynecological examination was performed for female participants.

### Laboratory measurements

Participants underwent a series of laboratory tests to evaluate hematological, metabolic, hepatic, renal, and urinary parameters, as well as tumor biomarkers. All tests followed standardized clinical protocols to ensure accuracy and reproducibility.

Routine blood, biochemical, and urine tests were conducted at the laboratory departments of community health service centers, using automated analyzers. Hematological tests included complete blood counts (CBC), measuring hemoglobin (g/L), white blood cell count (10⁹/L), red blood cell count (10⁹/L), platelet count (10⁹/L), lymphocyte count (10⁹/L), lymphocyte percentage, mean corpuscular volume (fL), mean corpuscular hemoglobin (pg), mean corpuscular hemoglobin concentration (g/L), mean platelet volume (fL), and platelet distribution width (fL). Hematology was measured on an automated analyzer XN-1000 (Sysmex, Kobe, Japan/Shanghai, China) using fluorescence flow cytometry and DC detection with hydrodynamic focusing; hemoglobin was quantified by the SLS method. Metabolic assessments included fasting blood glucose (FBG, mmol/L) and lipid profiles, consisting of total cholesterol (TC, mmol/L), triglycerides (TG, mmol/L), high-density lipoprotein cholesterol (HDL-C, mmol/L), and low-density lipoprotein cholesterol (LDL-C, mmol/L). Hepatic and renal function tests assessed organ health and metabolic function, including alanine aminotransferase (ALT, U/L), aspartate aminotransferase (AST, U/L), creatinine (µmol/L), urea (mmol/L), and uric acid (µmol/L). Serum chemistry (fasting glucose, lipid profile, liver/kidney function) was measured on an automated analyzer Polarisc2000 (KHB, Shanghai, China) using photometric end-point/rate methods. Urinalysis was performed to detect urine glucose, urine ketones, urine protein, and urine occult blood with an automated urine chemistry analyzer Mejer-700I (Meiqiao, Shenzhen, China) by reflectance photometry.

In addition, tumor biomarker tests were conducted on a subset of participants in specialized laboratories. Alpha-fetoprotein (AFP) and hepatitis B surface antigen (HBsAg) were assayed by ELISA (Autobio, China), as well as Epstein-Barr virus viral capsid antigen immunoglobulin A (EBV VCA-IgA) by ELISA (EUROIMMUN, Germany), all following the manufacturers’ instructions. Carcinoembryonic antigen (CEA), a widely used tumor marker for gastrointestinal and lung cancers, was measured by radioimmunoassay (RIA) at Guangzhou KingMed Diagnostics (CAP-accredited).

To maintain quality control, each laboratory followed internal calibration protocols, conducted regular proficiency testing, and implemented quality assurance procedures to minimize measurement variability. All test results were interpreted by certified laboratory physicians, ensuring clinical validity and data reliability.

### Auxiliary examinations

Auxiliary diagnostic assessments were conducted to further evaluate participants’ health status. These examinations were performed by trained specialists following standardized clinical protocols. A standard 12-lead resting electrocardiogram (ECG) was recorded using an ECG-1112 M electrocardiograph (Shenzhen Kaiwo Electronics Co., Ltd., Shenzhen, China). Chest X-rays were obtained with an MXHF-1500DR digital radiography system (Beijing Zhongji Guobei Medical Technology Co., Ltd., Beijing, China). Abdominal ultrasonography was performed using an HS50 ultrasound system (Samsung Medison Co., Ltd., Seoul, South Korea) to evaluate the liver, gallbladder, pancreas, spleen, and kidneys. For female participants, pelvic ultrasonography was conducted with the same system, along with cervical screening including Pap smear and pathological examination where clinically indicated.

### Biospecimen collection and biological biobank

To support multi-omics analyses and biomarker discovery, fasting blood and saliva samples were systematically collected from all participants at baseline. Blood samples (10 mL) were drawn after overnight fasting (≥ 8 h) using EDTA anticoagulant tubes. Following centrifugation at 3,500 rpm for 10 min, the plasma, buffy coat, and red blood cell fractions were separated and aliquoted into labeled tubes. Saliva samples (3 mL) were self-collected after fasting (no food or water intake) into collection tubes pre-filled with a stabilization buffer to preserve nucleic acids and microbial composition. For each participant, the blood samples were divided into five aliquots, including three tubes of plasma, one tube of buffy coat (leukocytes), and one tube of red blood cells, while saliva samples were divided into three aliquots. Each sample was assigned a unique participant ID and linked to a QR code, which was scanned into the biobank management system for real-time tracking of sample location, volume, and processing details.

All processed samples were stored in − 80 °C freezers. All these freezers are equipped with real-time temperature monitoring and alarm systems, with alerts triggered when the temperature exceeds a predefined threshold (typically − 70 °C). Freezers are linked to a centralized system, and routine inspections are performed under SOPs. The power supply is configured with a dual-circuit redundant system, ensuring uninterrupted operation during emergency scenarios. To ensure high-quality biospecimens, the entire process—from sample collection to aliquoting and storage at − 80 °C—was completed within six hours. Rigorous quality control protocols were implemented, including integrity assessments in randomly selected samples, confirming their suitability for genomics, transcriptomics, epigenomics, proteomics and microbiomics research. The GDBC biobank currently houses over 400,000 aliquots, providing a valuable resource for future studies.

### Multi-omics sub-cohort

the first phase of the study prioritized multi-omics profiling in a sub-cohort to investigate the genetic and microbial contributions to disease susceptibility and their interactions with environmental and lifestyle factors.

A subset of 2,530 participants underwent genome-wide genotyping using the Illumina Infinium Global Screening Array-24 Kit. Genomic DNA was extracted from buffy coat fractions of blood samples following standardized protocols. Quality control (QC) procedures were applied at both variant and individual levels. Variants with low call rates (< 95%), minor allele frequency (MAF) < 0.01, or significant deviations from Hardy-Weinberg equilibrium (*P* < 10⁻⁷ in controls or *P* < 10⁻¹² in cases) were excluded. Individuals with gender discrepancies, high variant missing rates (< 95%), extreme heterozygosity (>6 SD), cryptic familial relatedness (PI_HAT >0.25), or population outliers (determined via PCA using EIGENSTRAT) were removed. To enhance genotype resolution, imputation analyses were performed using different methods for MHC and non-MHC regions. For non-MHC regions, phasing was conducted with SHAPEIT (v2.12), and imputation was performed using IMPUTE2, with the 1000 Genomes Phase III dataset as the reference panel. For MHC regions, SNP2HLA was used with the Han Chinese reference panel (BGI, *n* = 10,689). Variants with low imputation quality or abnormal allele frequencies were removed [31].

To evaluate population structure and genetic homogeneity, we conducted principal component analysis (PCA) on high-quality autosomal SNPs after linkage disequilibrium (LD) pruning with PLINK (v1.9) (window size 200 SNPs, step 50 SNPs, r² = 0.1). Runs of homozygosity (ROH) were estimated using PLINK (v1.9) (minimum length = 1 Mb, ≥ 50 SNPs). Genomic control (λ_GC) analysis was performed by conducting genome-wide association analyses on (i) a randomly simulated phenotype and (ii) sex, with λGC calculated from the median χ² statistic of autosomal SNPs.

To explore the role of the oral microbiome in disease development and its interactions with host genetic and environmental factors, 16 S rRNA sequencing was performed on 2,049 participants. Saliva samples were collected in preservative-filled tubes, and microbial DNA was extracted using the PowerSoil DNA Isolation Kit (Qiagen, Germany). The V4 region of the 16 S rRNA gene was amplified using primer pairs 515 F/806R and sequenced on an Illumina MiSeq platform (2 × 250 bp paired-end reads). Raw sequencing data were demultiplexed based on sample-specific barcodes, and quality control and denoising were conducted using DADA2 to generate amplicon sequence variants (ASVs). Microbial composition and diversity were analyzed using the QIIME2 pipeline, enabling taxonomic classification, alpha and beta diversity analysis, and microbial community profiling [32].

All multi-omics data underwent rigorous quality control and preprocessing to ensure data integrity and reproducibility. This multi-omics sub-cohort provides a foundation for future large-scale analyses, supporting research into gene-environment interactions, host-microbiome interplay, and complex disease mechanisms.

## How is the cohort followed up?

### Dynamic monitoring subset (Phase I)

To investigate longitudinal changes in lifestyle factors, physiological indicators, and disease biomarkers, a dynamic monitoring subset (Phase I) was established within the GDBC cohort. This sub-cohort aims to track risk factor progression and early disease markers through periodic follow-ups, providing insights into the development of NCD risk factors and their long-term health impact.

Following the baseline survey, approximately 44.18% of participants from each community health service center were invited for a follow-up resurvey conducted between 2020 and 2022, with plans for subsequent resurveys at three- to five-year intervals. Physical examinations, auxiliary assessments, and biospecimen collection adhered to the same standardized protocols as the baseline survey, ensuring consistency in measurements. These included anthropometric assessments, head and neck examinations, abdominal ultrasound imaging, and gynecological examinations for female participants. Fasting blood and saliva samples were collected, and laboratory tests, including routine blood test, fasting blood glucose, lipid profiles, hepatic and renal function tests, were performed in the same community health service center laboratories as at baseline.

This dynamic follow-up strategy enhances the cohort’s ability to identify early physiological and biochemical changes, supporting research on NCD progression, early detection strategies, and potential interventions.

### Outcome follow-up and ascertainment

To ensure comprehensive tracking of disease incidence and mortality, the GDBC cohort employs a combination of active and passive follow-up methods. Passive follow-up is conducted through linkage with electronic health records (EHRs), cancer registries, chronic disease surveillance systems, and death registries maintained by local health authorities. The cohort is integrated with the Zhongshan City Disease Surveillance System, which captures hospital admissions, outpatient visits, and cancer diagnoses using the ICD-10 classification system. To complement passive data collection, active follow-up is conducted through telephone interviews and in-person visits at community health service centers, targeting participants with incomplete health records or those who missed scheduled health check-ups. Trained personnel collect self-reported disease diagnoses, medication use, and hospital admissions. Main NCDs including hypertension, diabetes, and cancer, verified through medical record review and physician confirmation. These NCDs outcomes are defined based on standardized clinical criteria, with cancer incidence confirmed via the Zhongshan Cancer Registry and histopathological reports. All the participants were followed up annually from the recruitment until their death. For the identified NCD cases, detailed diagnosis and clinical data for all incident NCD cases were retrieved from the hospital electronic medical record. The follow-up time for NCDs was defined as the period from the baseline survey to the date of diagnosis for the relevant incident NCDs, or to the last follow-up date (Dec 30, 2023) for participants that did not develop any incident NCDs during the study period. This multi-source follow-up strategy ensures high accuracy and completeness of outcome ascertainment, enabling robust epidemiological analyses on disease progression, risk prediction, and long-term health outcomes.

## What has been found?

At baseline, a total of 35,081 participants were enrolled in the GDBC cohort, comprising 21,533 (61.38%) urban and 13,548 (38.62%) rural residents. The mean age was 57.64 ± 10.45 years, and 64.98% were female. The age distribution was relatively balanced, with the majority (72.83% vs. background population: 71.38%) aged between 45 and 69 years (Table 2). 42.96% of participants completed middle education, while 33.61% had only primary school education and below. 77.34% were married, with relatively higher proportions in urban residents than rural areas (83.28% vs. rural: 67.90%). Only a small fraction were single (1.54%), divorced (4.27%), or widowed (1.26%).

**Table 2:** The characteristics of the Guangdong biobank cohort subjects at baseline

Variable	ALL	Urban (*n* = 21533)	Rural (*n* = 13548)
	*N* (%)	*N* (%)	*N* (%)
*Age, years*	57.64 ± 10.45	57.78 ± 10.45	57.41 ± 10.45
40–44	4466 (12.73)	2680 (12.45)	1786 (13.18)
45–49	4966 (14.16)	3046 (14.15)	1920 (14.17)
50–54	4849 (13.82)	2876 (13.36)	1973 (14.56)
55–59	5062 (14.43)	3177 (14.75)	1885 (13.91)
60–64	5410 (15.42)	3243 (15.06)	2167 (15.99)
65–69	5263 (15.00)	3346 (15.54)	1917 (14.15)
70–74	3321 (9.47)	2083 (9.67)	1238 (9.14)
75–79	1332 (3.80)	844 (3.92)	488 (3.60)
80-84	412 (1.17)	238 (1.11)	74 (1.28)
*Sex, n (%)*			
Male	12,286 (35.02)	7607 (35.33)	4679 (35.54)
Female	22,795 (64.98)	13,926 (64.67)	8869 (65.46)
*Educational level, n (%)*			
Primary school and below	11,793 (33.61)	7832 (36.37)	3961 (29.24)
Middle school	15,069 (42.96)	10,222 (47.47)	4847 (35.77)
College and above	3558 (10.14)	1462 (6.79)	2096 (15.47)
Unknown/NA	4661 (13.29)	2017 (9.37)	2644 (19.52)
*Marital status, n (%)*			
Single	540 (1.54)	219 (1.02)	321 (2.43)
Married	27,132 (77.34)	19,733 (83.28)	9199 (67.90)
Divorced	1499 (4.27)	524 (2.43)	975 (7.20)
Widowed	443 (1.26)	338 (1.57)	105 (0.78)
Unknown/NA	5467 (15.58)	2519 (11.70)	2948 (21.76)
*Cigarette smoking, n (%)*			
Non-Smoker	25,947 (73.96)	16,963 (78.78)	8984 (66.31)
Current smoker	4413 (12.58)	2594 (12.05)	1819 (13.43)
Former Smoker	1923 (5.48)	1195 (5.55)	728 (5.37)
Unknown/NA	2798 (7.98)	781 (3.63)	2017 (14.89)
*Alcohol consumption, n (%)*			
Non-drinker	25,936 (73.93)	17,005 (78.97)	8931 (65.92)
Occasionally	3169 (9.03)	1992 (9.25)	1177 (8.69)
Frequently	1266 (3.61)	802 (3.72)	464 (3.42)
Everyday	1901 (5.42)	949 (4.41)	952 (7.02)
Unknown/NA	2809 (8.01)	785 (3.65)	2024 (14.94)
*Exercise frequency, n (%)*			
None	9366 (26.70)	5165 (23.99)	4201 (31.01)
Occasionally	5246 (14.95)	2807 (13.04)	2439 (18.00)
Frequently	4323 (12.32)	3401 (15.79)	922 (6.81)
Everyday	12,330 (35.15)	8753 (40.65)	3577 (26.40)
Unknown/NA	3816 (10.88)	1407 (6.53)	2409 (17.78)
*Body mass index (BMI)*			
18.5–22.9	12,483 (35.58)	8150 (37.85)	4333 (31.98)
< 18.5	1314 (3.75)	868 (4.03)	446 (3.29)
23.0–27.5	14,951 (42.62)	9244 (42.93)	5707 (42.12)
> 27.5	4731 (13.49)	2798 (12.99)	1933 (14.27)
Unknown/NA	1602 (4.57)	473 (2.20)	1129 (8.33)
*Physical examination, mean ± SD*			
Height, cm	159.06 ± 11.92	159.44 ± 13.73	158.46 ± 7.93
Weight, kg	60.65 ± 10.49	60.60 ± 10.62	60.74 ± 10.28
Body mass index	23.92 ± 3.37	23.80 ± 3.36	24.13 ± 3.39

Regarding lifestyle behaviors, 73.96% were non-smokers, 12.58% were current smokers and 5.48% were former smokers. The smoking prevalence was slightly higher among rural participants (13.43%) than urban participants (12.05%). Similarly, 73.93% of participants reported no alcohol consumption, while 5.42% drank daily. Daily alcohol consumption was more common in rural areas (7.02%) than in urban areas (4.41%). In terms of physical activity, 35.15% of participants reported exercising daily, while 26.70% reported no regular exercise. Urban residents reported more favorable health behaviors. About 40.65% of urban participants exercised daily compared to 26.40% in rural areas. Conversely, 31.01% of rural participants reported no regular exercise, significantly higher than 23.99% in urban areas (Table 2). Anthropometric and clinical measurements showed that urban residents had lower waist and hip circumferences, lower blood pressure, fat mass, and improved lipid profiles compared to rural residents, including lower triglycerides and LDL-C, and higher HDL-C (Table S2).

PCA of the genotyped sub-cohort revealed a single dense cluster without evidence of substructure, with top components explaining about 6% of variance, and variance explained decreased gradually across subsequent components (Fig. S2). ROH analysis showed consistent homozygosity levels across individuals, with no outliers indicative of recent inbreeding (Fig. S3). Genomic control analyses showed no inflation (λGC = 0.998 for simulated phenotype; λGC = 1.01 for sex), indicating negligible population stratification (Fig. S4).

**Fig. 2:**
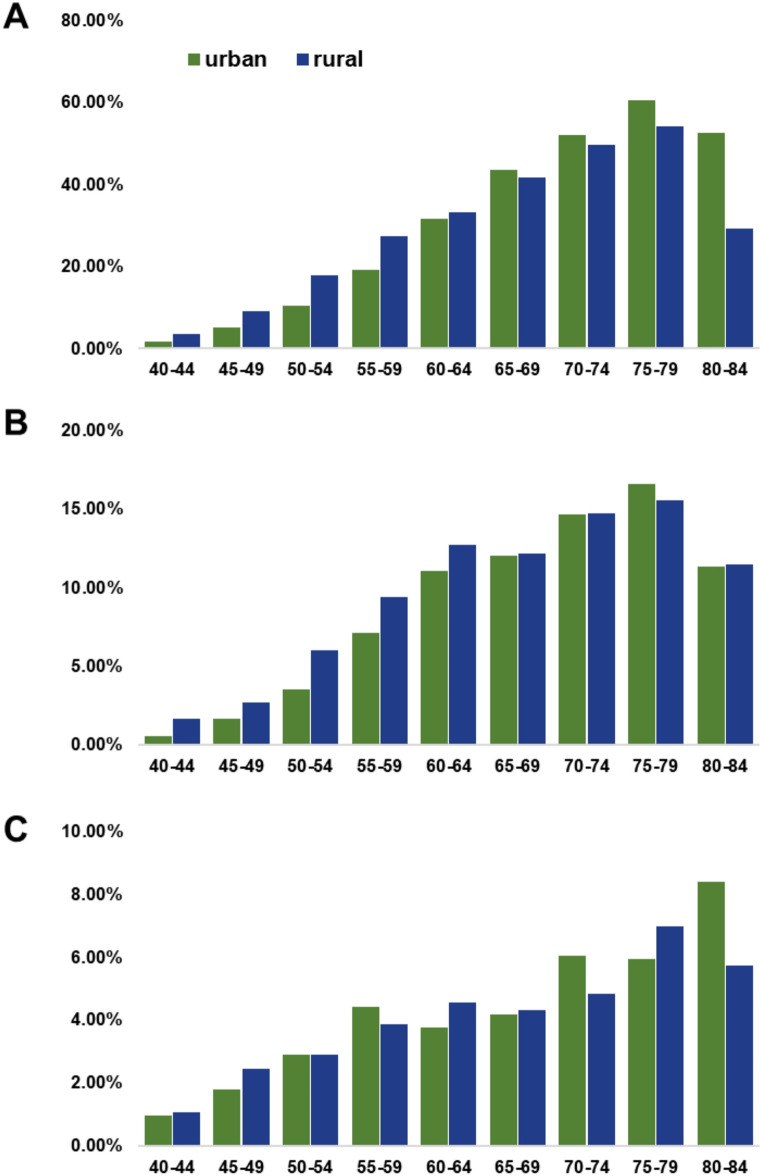
Age distribution of the prevalent cases of hypertension (**A**), diabetes (**B**), and cancer (**C**) at baseline, stratified by rural and urban residence

**Fig. 3:**
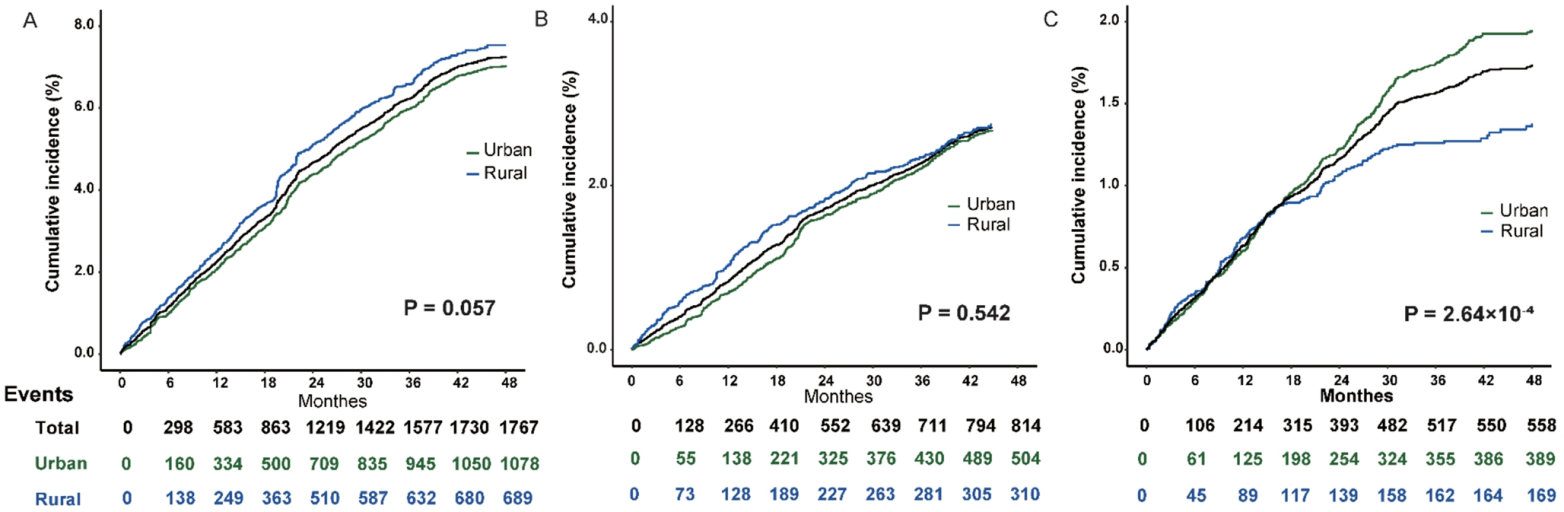
Incident cases of hypertension (**A**), diabetes (**B**), and cancer (**C**) during follow-up, stratified by rural and urban residence. The p-values for urban–rural differences were calculated using the log-rank test

At baseline, hypertension was one of the most prevalent NCDs condition, affecting 8,879 participants with a crude prevalence of 25.31%. Prevalence was higher in rural residents than in urban (age-sex standardized prevalence [ASP]: 22.56% vs. 18.94%, Table S3). Age-specific analysis revealed that rural participants had higher prevalence in younger age groups (< 65 years), but this trend reversed in those aged ≥ 65 years (Fig. 2A). During follow-up through December 2023, 1,767 incident hypertension cases occurred among 26,202 participants, with a crude incidence of 1,804.64/100,000 person–years (Table S3). The cumulative incidence was marginally higher in rural areas compared to urban (age-sex standardized incidence [ASI]: 1,860.47 vs. 1,500.63 per 100,000 person-years, *P* = 0.057, Fig. 3A). Diabetes was reported in 2,788 participants (7.95%) at baseline, with a slightly higher prevalence in rural areas than urban areas (ASP:7.62% vs. 5.97%, Table S3). The rural-urban disparity was more pronounced among individuals younger than 64 years, while it narrowed in older age groups (Fig. 2B). During follow-up, 814 incidence diabetes cases were recorded, with a crude incidence of 2.52%, and no significant urban-rural difference was observed (*P* = 0.542, Fig. 3B). Cancer history was reported in 1,244 participants (3.55%) at baseline, with similar prevalence in urban and rural groups (ASP: 3.00% vs. 2.95%, Table S3). During the follow-up, 558 incident cancer cases occurred, corresponding to a crude incidence of 357.32/100,000 person-years. The incidence was significantly higher in urban areas than in rural areas (ASI: 377.76 vs. 310.53 per 100,000 person-years, *P* = 2.64 × 10⁻⁴; Fig. 3C).

Cox regression analyses indicated that older age (≥ 60 years) was consistently associated with higher risk of hypertension (HR = 2.83, 95% CI: 2.52–3.17; *P* < 0.001), diabetes (HR = 1.89, 1.60–2.23; *P* < 0.001), and cancer (HR = 1.49, 1.19–1.87; *P* < 0.001), consistent with global age-related patterns reported by the Global Burden of Disease collaboration [33, 34]. Rural residence was significantly associated with increased hypertension risk (HR = 1.17, 95% CI: 1.04–1.32, *P* = 0.01), but not with diabetes (HR = 1.12, 0.95–1.32) or cancer (HR = 0.85, 0.66–1.08). This rural ≥ urban pattern resembles high-income settings such as the United States [35, 36], whereas many low- and middle-income countries show higher urban prevalence [36]. Current smoking (HR = 1.24, 95% CI: 1.04–1.48, *P* = 0.01) and daily alcohol consumption (HR = 1.27, 95% CI: 1.02–1.57, *P* = 0.03) were associated with higher hypertension risk, in line with international studies [37, 38]. For total cancer, the smoking association was not statistically significant (HR = 1.23, 95% CI: 0.86–1.78; *P* = 0.26), which may reflect the relatively short follow-up and limited number of incident cases, the low prevalence of active smoking, and cancer-site heterogeneity (e.g., undifferentiated nasopharyngeal carcinoma) [39, 40]. Higher BMI (>27.5 kg/m²) was strongly associated with increased risk of hypertension and diabetes: HRs of 2.00 (95% CI: 1.76–2.26; *P* < 0.001) for BMI 23.0–27.5 and 2.80 (2.38–3.29; *P* < 0.001) for BMI >27.5 for hypertension; and 2.57 (2.07–3.18; *P* < 0.001) and 5.50 (4.37–6.92; *P* < 0.001), respectively, for diabetes (Table S4). These results were consistent with findings from large international cohorts such as the UK Biobank and the U.S. National Health and Nutrition Examination Survey (NHANES) [41, 42].

### Key findings and publications

Using data from sub-cohorts of the GDBC and multi-center collaborations, we identified novel genetic, microbial, and viral biomarkers to improve cancer risk stratification and early detection in high-incidence regions of Southern China.

For NPC and lung cancer, we conducted genome-wide association studies (GWAS) and identified several common susceptibility variants. We developed a polygenic risk score (PRS) derived from these variants, which significantly improved NPC risk prediction, particularly when combined with EBV serology [31, 43]. Based on the identified NPC-related susceptibility variants, further functional analyses identified *PHF2* and *CDKN2B-AS1* as regulatory mediators of oncogenic pathways [44]. For lung cancer, 19 risk loci, including six novel variants, were identified, and the derived PRS demonstrated predictive performance independent of age and smoking in prospective cohort validation [45].

Through oral microbiome profiling, we found that dysbiosis of heritable and transmissible taxa was closely associated with NPC risk [46]. In addition, we observed that lifestyle factors such as poor oral hygiene, smoking, and alcohol consumption, were associated with altered microbial composition and metabolic profiles, potentially promoting disease through microbial-mediated mechanisms [47–49].

At the population level, we evaluated and optimized screening strategies for NPC. A population-based randomized trial demonstrated that EBV serological screening significantly improved early detection and reduced NPC-specific mortality, particularly among individuals aged ≥ 50 years [50, 51]. To improve diagnostic performance, novel antibody biomarkers, such as anti-BNLF2b (P85-Ab), were developed and shown to outperform traditional EBV markers in terms of sensitivity, specificity, and positive predictive value [52, 53]. Our gene-virus interaction analyses indicated that the effect of genetic susceptibility to NPC is substantially mediated or modified by EBV serological response, supporting the implementation of host-virus integrated models for precision screening [54]. Integration of polygenic risk score into EBV-based screening further improved cost-effectiveness and reduced unnecessary procedures [55].

We also developed and validated multiple EBV-related biomarkers for non-invasive and potentially home-based NPC screening. We found that EBV DNA methylation markers in saliva samples offered strong diagnostic performance across various oral sample types, enabling self-collection outside clinical settings [56]. Moreover, we developed a CRISPR/Cas12a-based digital assay that significantly outperformed conventional qPCR in detecting plasma EBV DNA, especially in early-stage NPC, offering improved sensitivity and quantitative precision [57].

Beyond NPC, we found that elevated EBV antibody levels were positively associated with increased risks of gastric and liver cancers [58, 59]. Notably, we observed a strong synergistic effect between EBV and hepatitis B virus (HBV) seropositivity in liver cancer development [59]. However, liver cancer screening based on current national guidelines—although effective in early detection—did not significantly reduce short-term mortality, highlighting the need for more targeted and risk-adapted strategies [60]. Finally, we validated a novel plasma glycoprotein biomarker (ofCS-CD44) with pan-cancer detection potential. Individuals in the top decile of this marker showed a >27-fold increased cancer risk, suggesting promise for early detection across multiple tumor types [61].

## What are the main strengths and weakness?

As a regionally representative cohort in Southern China, the GDBC has several strengths. First, the GDBC established a population-based cohort of Guangdong Southern Chinese residents—a population with distinct genetic and geographic backgrounds, which was underrepresented in existing national cohorts. Given China’s substantial regional variations in genetic background [62], lifestyle, socioeconomic status, and environmental exposures, the GDBC offers valuable insights into the etiology and prevention of chronic diseases in this population. Second, by leveraging a cloud-based digital platform, the study encompassed comprehensive individual information including environmental exposure, dietary habits, lifestyle factors, physical measurements, and biochemical parameters from blood and urine laboratory test. These are systematically linked to regional disease surveillance registries, enabling systematic analysis for the etiology in NCDs and facilitating identification of modifiable risk factors for NCDs prevention. Third, the GDBC integrated multi-omics data, including genome-wide variants, oral microbiota profiles and metabolic biomarkers (such as blood glucose, lipid profile etc.), allowing for the exploration of genetic-microbe-environment interactions in NCDs pathogenesis [63, 64]. These multidimensional data frameworks could facilitate high-resolution mechanistic dissection of host-microbial crosstalk, driving the investigation of personalized prevention strategies. Fourth, the samples and data collected from the ongoing dynamic monitoring subsets could potentially capture temporal epigenetic or metabolic changes during pre-symptomatic transitions before clinical onset of NCDs, providing abundant longitudinal resources for predictive modeling of NCDs, and developing potential prophylactic approaches targeting pre-disease. Fifth, the construction and follow-up of GDBC is sustained funding from China’s National Key R&D Program and municipal health investment annually, providing sustainable and longitudinal resources for prevention and translational research of NCDs. However, a few limitations of GDBC should also be considered. The cohort exhibits an underrepresentation of middle-aged men (especially those aged 40–60 years), which possibly leads to selection bias. This socioeconomic selection bias likely originates from regional labor migration patterns from tier 3 cities to metropolitan hubs, as documented in China’s 2020 Population Census [65]. Secondarily, Since the cohort members primarily participated on a voluntary basis after receiving invitations, self-selection bias may exist. However, the baseline prevalence of major NCDs in our cohort is consistent with estimates from previous researches [66, 67] and regional chronic disease surveillance data [68]. In addition, association analyses revealed that the relationships between lifestyle factors and NCDs were reasonable and consistent with findings from previous population-based studies [38, 69–71], indicating that the representativeness of the cohort population would not be a major concern.

## Can I get hold of the data? Where can I find out more?

In compliance with medical ethical principles and data privacy protection guidelines, access to the GDBC data and biospecimens is restricted to approved researchers within the project team and institutional collaborators. All data use is managed under controlled requiring formal ethical and scientific review for access. The raw data of the baseline characteristics of the GDBC has been deposited in the Research Data Deposit platform (www.researchdata.org.cn, accession code: RDDA2025796503). However, the GDBC welcomes and encourages international research collaboration to amplify utility of the multidimensional research data. To initiate a collaboration, interested researchers should contact Prof. Wei-Hua Jia and Yong-Qiao He. The contact information for the administrator is [jiawh@sysucc.org.cn/heyq@ sysucc.org.cn].

## Supplementary Information

Below is the link to the electronic supplementary material.

Supplementary Material 1
