# Supplementary material for: Guangdong Biobank Cohort (GDBC) study
Source: Eur J Epidemiol. 2026 Jan 13;41(2):229–43. doi: 10.1007/s10654-025-01320-y (PMC12975853; doi:10.1007/s10654-025-01320-y)

**Journal name: European Journal of Epidemiology**

**Cohort profile: Guangdong Biobank Cohort (GDBC) Study**

**Supplementary Materials**

**Authors**

Yong-Qiao He^1,* ,#^, Wen-Qiong Xue^1,*^, Hua Diao^2,3*^, Ji-Yun Zhan^4,*^, Ming-Fang Ji^5,*^ Da-Wei Yang^2,6^, Yi Zhao^2^, Chang-Mi Deng^1,7^, Zi-Yi Wu^1,8^, Ting Zhou^1^, Ying Liao^1^, Mei-Qi Zheng^1^, Wen-Li Zhang^1,7^, Yi-Jing Jia^2^, Lei-Lei Yuan^2,9^, Lu-Ting Luo^2,10^, Dan-Hua Li^1,11^, Tong-Min Wang^1^, Xia-Ting Tong^2,12^, Yan Du^1^, Ling-Ling Tang^1,6^, Jing-Wen Huang^2^, Chang-ling Huang^2^, Zhi-Yang Zhao^2^, Yan-Xia Wu^1^, Lian-Jing Cao^1,13^ Si-Qi Dong^1,7^, Fang Wang^1,14^, Cheng-Tao Jiang^1^, Ruo-Wen Xiao^1,15^, Wen-Bin Zhang^1,16^ Xue-Yin Chen^1^, Qiao-Ling Wang^2,17^, Qiao-Yun Liu^2^, Yue-Ze Zhao^2^, Cao-Li Tang^2^, Lin Ma^2^, Xiao-Hui Zheng^1^, Pei-Fen Zhang^1^, Xi-Zhao Li^1^, Shao-Dan Zhang^1^, Ye-Zhu Hu^1^, Xia Yu^5^, Biao-Hua Wu^5^, Fu-Gui Li^5^, Jian-Hua Wu^4^, Bi-Sen Deng^4^, Xue-Jun Liang^4^, Wei-Hua Jia^1,2,#^

**Affiliations**

1. State Key Laboratory of Oncology in South China, Collaborative Innovation Center for Cancer Medicine, Guangdong Key Laboratory of Nasopharyngeal Carcinoma Diagnosis and Therapy, Sun Yat-sen University Cancer Center, Guangdong, P. R. China;
2. School of Public Health, Sun Yat-Sen University, Guangdong, P. R. China;
3. Department of Gastroenterology, Third Military Medical University Xinqiao Hospital, Chongqing, P.R. China;
4. Public Health Service Center of Xiaolan Town, Guangdong, P. R. China;
5. Cancer Research Institute of Zhongshan City, Zhongshan City People’s Hospital, Zhongshan, Guangdong, P. R. China;
6. Guangdong Provincial Center for Disease Control and Prevention, Guangdong, P. R. China;
7. Guangzhou Women and Children's Medical Center, Guangzhou Medical University, Guangdong, P. R. China;
8. Clinical Oncology School of Fujian Medical University, Fujian Cancer Hospital, Fujian, P. R. China;
9. Shenzhen Maternity and Child Healthcare Hospital, Southern Medical University, Shenzhen, Guangdong, P. R. China;
10. Shenzhen Futian Center for Chronic Disease Control, Guangdong, P. R. China;
11. Foshan Key Laboratory of Precision Therapy in Oncology and Neurology, Department of Pulmonary Oncology, The First People’s Hospital of Foshan, Guangdong, P. R. China;
12. Zengcheng District Center for Disease Control and Prevention, Guangdong, P. R. China;
13. Gastrointestinal Cancer Institute/Pancreatic Disease Institute, the Affiliated Hospital of Qingdao University, Qingdao, P. R. China;
14. Department of Radiation Oncology, Affiliated Cancer Hospital & Institute of Guangzhou Medical University, Guangdong, P. R. China;
15. Department of Oncology, The First Hospital of Lanzhou University, Gansu, P.R. China;
16. Department of Urology, The First Affiliated Hospital of Guangzhou Medical University Guangdong, Guangdong, P. R. China;
17. The Eighth Affiliated Hospital of Sun Yat-sen University, Guangdong, P. R. China;

* These authors have contributed equally to this work.

# Corresponding author to: Wei-Hua Jia, M.D., Ph.D.

Professor, State Key Laboratory of Oncology in South China, Collaborative Innovation Center for Cancer Medicine, Sun Yat-Sen University Cancer Center, 651 Dong Feng East Road, Guangzhou, Guangdong 510060, China,

Tel.: (+86-20) 87342327; E-mail: [jiawh@sysucc.org.cn](mailto:jiawh@sysucc.org.cn)

Yong-Qiao He, M.D., Ph.D., State Key Laboratory of Oncology in South China, Collaborative Innovation Center for Cancer Medicine, Sun Yat-Sen University Cancer Center, 651 Dong Feng East Road, Guangzhou, Guangdong 510060, China,

Tel.: (+86-20) 87342410; E-mail: heyq@sysucc.org.cn

**Table S1 Age distribution of the subjects in Guangdong Biobank cohort compared to the registered population in Xiaolan Town**

| **Age（year）** | **Males** | | | **Females** | | | **Total** | | | |
| --- | --- | --- | --- | --- | --- | --- | --- | --- | --- | --- |
|  | Background population ^a^ | Cohort subject | Coverage | Background population ^a^ | Cohort subject | Coverage | Background population ^a^ | Cohort subject | Coverage |  |
| 40～44 | 5988 | 1062 | 17.74% | 6440 | 3404 | 52.86% | 12428 | 4466 | 35.93% |  |
| 45～49 | 6964 | 1380 | 19.82% | 7211 | 3586 | 49.73% | 14175 | 4966 | 35.03% |  |
| 50～54 | 6934 | 1471 | 21.21% | 7034 | 3378 | 48.02% | 13968 | 4849 | 34.72% |  |
| 55～59 | 4781 | 1502 | 31.42% | 4900 | 3560 | 72.65% | 9681 | 5062 | 52.29% |  |
| 60～64 | 5148 | 2393 | 46.48% | 5452 | 3017 | 55.34% | 10600 | 5410 | 51.04% |  |
| 65～69 | 3725 | 2405 | 64.56% | 4365 | 2858 | 65.48% | 8090 | 5263 | 65.06% |  |
| 70～74 | 2012 | 1371 | 68.14% | 2703 | 1950 | 72.14% | 4715 | 3321 | 70.43% |  |
| 75～79 | 1371 | 548 | 39.97% | 1968 | 784 | 39.84% | 3339 | 1332 | 39.89% |  |
| 80～84 | 879 | 154 | 17.52% | 1297 | 258 | 19.89% | 2176 | 412 | 18.93% |  |
| Total | 37802 | 12286 | 32.50% | 41370 | 22795 | 55.10% | 79172 | 35081 | 44.31% |  |

^a^ Registered population in Xiaolan in 2016

**Table S2 The distribution of parameters in baseline physical examination and blood laboratory tests for the cohort subjects**

| **Variable** | **ALL** | **Urban (n=21533)** | **Rural (n=13548)** |
| --- | --- | --- | --- |
|  | Mean ± SD | Mean ± SD | Mean ± SD |
| **Physical examination parameters** |  |  |  |
| Waist circumference (cm) | 83.22 ± 9.53 | 82.35 ± 9.60 | 84.66 ± 9.23 |
| Hip circumference (cm) | 94.29 ± 7.38 | 93.89 ± 7.46 | 94.93 ± 7.21 |
| Heart rate (per minute) | 75.21 ± 10.44 | 74.87 ± 9.48 | 75.83 ± 11.94 |
| Breath count (per minute) | 18.91 ± 1.29 | 18.85 ± 1.31 | 19.01 ± 1.26 |
| Grip strength (kg) | 26.96 ± 13.28 | 27.44 ± 13.28 | 26.20 ± 13.24 |
| Left: Systolic blood pressure, mmHg | 129.06 ± 17.39 | 128.86 ± 16.78 | 129.41 ± 18.43 |
| Left: Diastolic blood pressure, mmHg | 77.97 ± 10.13 | 77.66 ± 9.56 | 78.51 ± 11.06 |
| Right: Systolic blood pressure, mmHg | 130.04 ± 17.30 | 128.52 ± 16.65 | 132.73 ± 18.09 |
| Right: Diastolic blood pressure, mmHg | 78.58 ± 10.14 | 77.18 ± 9.50 | 81.05 ± 10.75 |
| Body fat percent (%) | 26.01 ± 7.39 | 25.62 ± 7.33 | 26.70 ± 7.45 |
| Visceral fat percent (%) | 9.50 ± 3.33 | 9.41 ± 3.33 | 9.67 ± 3.33 |
| Muscle percent (%) | 70.00 ± 7.18 | 70.39 ± 7.10 | 69.32 ± 7.28 |
| Bone mass percent (%) | 2.39 ± 0.41 | 2.40 ± 0.42 | 2.38 ± 0.39 |
| Water percent (%) | 46.91 ± 6.83 | 47.14 ± 6.98 | 46.51 ± 6.55 |
| Skeletal muscle percent (%) | 40.61 ± 5.43 | 40.81 ± 5.43 | 40.31 ± 5.43 |
| Subcutaneous fat percent (%) | 29.75 ± 7.99 | 29.33 ± 7.87 | 30.47 ± 8.14 |
| Basic metabolism (kcal/day) | 1339.17 ± 176.96 | 1345.65 ± 180.75 | 1327.79 ± 169.49 |
| **Routine blood test** |  |  |  |
| Hemoglobin, g/L | 141.14 ± 18.78 | 138.42 ± 15.79 | 145.71 ± 22.18 |
| Red blood cell count, 10^9^/L | 4.76 ± 0.60 | 4.72 ± 0.57 | 4.82 ± 0.65 |
| White blood cell count, 10^9^/L | 6.44 ± 1.82 | 6.17 ± 1.65 | 6.88 ± 2.00 |
| Platelet count, 10^9^/L | 228.36 ± 63.66 | 230.42 ± 62.83 | 224.93 ± 64.89 |
| Mean Corpuscular Volume, fL | 91.34 ± 9.22 | 90.19 ± 9.56 | 92.77 ± 8.57 |
| Mean Corpuscular Hemoglobin, pg | 30.02 ± 3.47 | 29.71 ± 3.01 | 30.42 ± 3.93 |
| Mean Corpuscular Hemoglobin Concentration, g/L | 327.76 ± 26.12 | 327.78 ± 19.60 | 327.72 ± 32.41 |
| Lymphocyte Absolute Count, 10^9^/L | 2.42 ± 1.46 | 2.48 ± 1.61 | 2.35 ± 1.26 |
| Lymphocyte Percentage, % | 33.71 ± 10.98 | 33.37 ± 11.28 | 34.63 ± 10.08 |
| Mean Platelet Volume, fL | 9.68 ± 2.20 | 9.59 ± 2.79 | 9.78 ± 1.22 |
| Platelet Distribution Width, fL | 12.86 ± 2.93 | 12.25 ± 2.52 | 13.61 ± 3.21 |
| **Fasting blood glucose, mmol/L** | 5.40±1.85 | 5.49±1.77 | 5.24±1.98 |
| **Hepatic function** |  |  |  |
| ALT, U/L | 23.38 ± 18.20 | 23.81 ± 19.03 | 22.64 ± 16.64 |
| AST, U/L | 24.52 ± 12.75 | 24.43 ± 13.12 | 24.66 ± 12.07 |
| ALT/AST | 0.97 ± 0.61 | 1.02 ± 0.72 | 0.89 ± 0.32 |
| Total bilirubin, umol/L | 12.61 ± 5.57 | 13.42 ± 5.73 | 11.06 ± 4.90 |
| **Renal function** |  |  |  |
| Creatinine, umol/L | 71.53 ± 24.98 | 70.02 ± 21.47 | 74.24 ± 30.05 |
| Urea, umol/L | 5.32 ± 1.58 | 5.23 ± 1.52 | 5.48 ± 1.68 |
| Uric acid, umol/L | 366.41 ± 98.61 | 365.22 ± 99.32 | 368.31 ± 97.46 |
| **Lipids** |  |  |  |
| TC, mmol/L | 5.49 ± 1.16 | 5.58 ± 1.16 | 5.32 ± 1.14 |
| TG, mmol/L | 1.61 ± 1.41 | 1.59 ± 1.31 | 1.67 ± 1.58 |
| HDL-C, mmol/L | 1.51 ± 0.50 | 1.58 ± 0.55 | 1.37 ± 0.38 |
| LDL-C, mmol/L | 3.30 ± 1.14 | 3.27 ± 1.02 | 3.34 ± 1.33 |

Abbreviation: ALT = Alanine aminotransferase. AST = Aspartate aminotransferase. HDL-C = High-density lipoprotein cholesterol. LDL-C = Low-density lipoprotein cholesterol. TC = Total cholesterol. TG = Triglycerides.

**Table S3 Outcomes of the Guangdong Biobank Cohort Study for the initial follow-up**

| Diseases | prevalent case | prevalent rate（%） | ASP (95% CI) ^a^  (%) | Participants  at risk | incident case | Crude incidence (/100,000 person-years) | ASI (95% CI) ^a^  (/100,000 person--years) |
| --- | --- | --- | --- | --- | --- | --- | --- |
| Hypertension | 8879 | 25.31 | 20.32 (19.84, 20.83) | 26202 | 1767 | 1804.64 | 1627.04 (1541.04, 1718.92) |
| Urban | 5317 | 24.69 | 18.94 (18.36, 19.55) | 16216 | 1078 | 1686.02 | 1500.63 (1400.38, 1611.15) |
| Rural | 3562 | 26.29 | 22.56 (21.69, 23.47) | 9986 | 689 | 2027.85 | 1860.47 (1698.46, 2043.19) |
| Diabetes | 2788 | 7.95 | 6.60 (6.31, 6.90) | 32293 | 814 | 649.72 | 572.31 (525.15, 625.12) |
| Urban | 1624 | 7.54 | 5.97 (5.63, 6.33) | 19909 | 504 | 515.83 | 535.83 (481.16, 598.67) |
| Rural | 1164 | 8.59 | 7.62 (7.10, 8.19) | 12384 | 310 | 706.75 | 656.19 (563.87, 769.04) |
| Cancer | 1244 | 3.55 | 2.97 (2.78, 3.17) | 33837 | 558 | 423.14 | 357.32 (323.55, 396.48) |
| Urban | 762 | 3.54 | 2.95 (2.72, 3.22) | 20771 | 389 | 456.60 | 377.76 (335.24, 428.20) |
| Rural | 482 | 3.56 | 3.00 (2.70, 3.35) | 13066 | 169 | 362.06 | 310.53 (258.26, 382.24) |

Abbreviation: ASP = Age-sex standardized prevalence. ASI = Age-sex standardized incidence. CI = Confident interval.

^a^: The age-sex standardized prevalence and incidence was calculated according to the 2010 China census population.

**Table S4 The association analysis between the baseline characteristics and risk of NCDs in the GDBC cohort**

| Variable | Hypertension | | | | Diabetes | | | | Cancer | | | |
| --- | --- | --- | --- | --- | --- | --- | --- | --- | --- | --- | --- | --- |
|  | Case (%) | Control (%) | HR (95%CI) | *P* value | Case (%) | Control (%) | HR (95%CI) | *P* value | Case (%) | Control (%) | HR (95%CI) | *P* value |
| Age, years |  |  |  |  |  |  |  |  |  |  |  |  |
| <60 | 709 (40.1) | 16417 (67.2) | 1.00 (reference) |  | 318 (39.1) | 18257 (58.0) | 1.00 (reference) |  | 204 (36.6) | 18639 (56.0) | 1.00 (reference) |  |
| >=60 | 1058 (59.9) | 8018 (32.8) | 2.83 (2.52, 3.17) | <0.001 | 496 (60.9) | 13222 (42.0) | 1.89 (1.60, 2.23) | <0.001 | 354 (63.4) | 14640 (44.0) | 1.49 (1.19, 1.87) | <0.001 |
| Rurality |  |  |  |  |  |  |  |  |  |  |  |  |
| Urban | 1078 (61.0) | 15138 (62.0) | 1.00 (reference) |  | 504 (61.9) | 19405 (61.6) | 1.00 (reference) |  | 389 (69.7) | 20382 (61.2) | 1.00 (reference) |  |
| Rural | 689 (39.0) | 9297 (38.0) | 1.17 (1.04, 1.32) | 0.01 | 310 (38.1) | 12074 (38.4) | 1.12 (0.95, 1.32) | 0.19 | 169 (30.3) | 12897 (38.8) | 0.85 (0.66, 1.08) | 0.18 |
| Sex, n (%) |  |  |  |  |  |  |  |  |  |  |  |  |
| Male | 665 (37.6) | 8128 (33.3) | 1.00 (reference) |  | 318 (39.1) | 10847 (34.5) | 1.00 (reference) |  | 232 (41.6) | 11631 (34.9) | 1.00 (reference) |  |
| Female | 1102 (62.4) | 16307 (66.7) | 1.08 (0.94, 1.25) | 0.28 | 496 (60.9) | 20632 (65.5) | 0.90 (0.74, 1.10) | 0.32 | 326 (58.4) | 21648 (65.1) | 1.06 (0.79, 1.42) | 0.68 |
| Educational level, n (%) |  |  |  |  |  |  |  |  |  |  |  |  |
| Primary school and below | 591 (40.2) | 7882 (37.3) | 1.00 (reference) |  | 289 (40.5) | 10529 (38.6) | 1.00 (reference) |  | 219 (44.6) | 11155 (38.7) | 1.00 (reference) |  |
| Middle school | 712 (48.4) | 10688 (50.5) | 1.00 (0.90, 1.12) | 0.97 | 348 (48.7) | 13590 (49.8) | 1.00 (0.85, 1.18) | 0.99 | 239 (48.7) | 14286 (49.5) | 0.99 (0.80, 1.01) | 0.92 |
| College and above | 167 (11.4) | 2583 (12.2) | 1.03 (0.83, 1.28) | 0.78 | 77 (10.8) | 3191 (11.7) | 1.15 (0.85, 1.56) | 0.37 | 33 (6.7) | 3420 (11.8) | 0.66 (0.39, 1.51) | 0.12 |
| Marital status, n (%) |  |  |  |  |  |  |  |  |  |  |  |  |
| Married | 1311 (91.3) | 18956 (91.9) | 1.00 (reference) |  | 647 (92.8) | 24389 (91.7) | 1.00 (reference) |  | 441 (93.6) | 25723 (91.6) | 1.00 (reference) |  |
| Single | 21 (1.5) | 368 (1.8) | 0.83 (0.54, 1.29) | 0.4 | 13 (1.9) | 490 (1.8) | 0.91 (0.52, 1.60) | 0.74 | 3 (0.6) | 521 (1.9) | 0.54 (0.17, 1.70) | 0.29 |
| Divorced | 83 (5.8) | 1006 (4.9) | 1.33 (1.01, 1.76) | 0.04 | 29 (4.2) | 1316 (4.9) | 0.81 (0.52, 1.26) | 0.35 | 17 (3.6) | 1434 (5.1) | 0.82 (0.38, 1.73) | 0.60 |
| Widowed | 21 (1.5) | 301 (1.5) | 0.83 (0.54, 1.29) | 0.41 | 8 (1.1) | 396 (1.5) | 0.73 (0.36, 1.48) | 0.39 | 10 (2.1) | 416 (1.5) | 1.48 (0.76, 2.89) | 0.24 |
| Cigarette smoking, n (%) |  |  |  |  |  |  |  |  |  |  |  |  |
| Non-Smoker | 1326 (75.6) | 18041 (82.5) | 1.00 (reference) |  | 605 (75.3) | 23323 (81.1) | 1.00 (reference) |  | 341 (73.0) | 24694 (80.4) | 1.00 (reference) |  |
| Current smoker | 312 (17.8) | 2779 (12.7) | 1.24 (1.04, 1.48) | 0.01 | 125 (15.6) | 3824 (13.3) | 1.10 (0.86, 1.42) | 0.45 | 84 (18.0) | 4227 (13.8) | 1.23 (0.86, 1.78) | 0.26 |
| Former Smoker | 116 (6.6) | 1047 (4.8) | 1.07 (0.85, 1.34) | 0.57 | 73 (9.1) | 1627 (5.7) | 1.18 (0.88, 1.58) | 0.28 | 42 (9.0) | 1788 (5.8) | 1.36 (0.88, 2.11) | 0.17 |
| Alcohol consumption, n (%) |  |  |  |  |  |  |  |  |  |  |  |  |
| Non-drinker | 1368 (78.0) | 17669 (80.8) | 1.00 (reference) |  | 620 (77.2) | 23158 (80.5) | 1.00 (reference) |  | 367 (78.6) | 24611 (80.2) | 1.00 (reference) |  |
| Occasionally | 169 (9.6) | 2280 (10.4) | 0.88 (0.73, 1.06) | 0.19 | 77 (9.6) | 2862 (10.0) | 0.88 (0.67, 1.15) | 0.34 | 38 (8.1) | 3060 (10.0) | 0.67 (0.43, 1.03) | 0.07 |
| Frequently | 72 (4.1) | 899 (4.1) | 1.08 (0.83, 1.41) | 0.57 | 33 (4.1) | 1136 (3.9) | 0.93 (0.62, 1.39) | 0.72 | 20 (4.3) | 1222 (4.0) | 1.09 (0.64, 1.85) | 0.76 |
| Everyday | 144 (8.2) | 1012 (4.6) | 1.27 (1.02, 1.57) | 0.03 | 73 (9.1) | 1607 (5.6) | 1.25 (0.93, 1.68) | 0.14 | 42 (9.0) | 1805 (5.9) | 1.09 (0.70, 1.70) | 0.69 |
| Exercise frequency, n (%) |  |  |  |  |  |  |  |  |  |  |  |  |
| None | 505 (29.0) | 6757 (32.3) | 1.00 (reference) |  | 240 (30.0) | 8493 (30.5) | 1.00 (reference) |  | 124 (29.4) | 8980 (30.2) | 1.00 (reference) |  |
| occasionally | 299 (17.2) | 3872 (18.5) | 1.08 (0.92, 1.27) | 0.35 | 115 (14.4) | 4760 (17.1) | 0.89 (0.70, 1.15) | 0.38 | 70 (16.6) | 5008 (16.8) | 1.14 (0.82, 1.59) | 0.43 |
| Frequently | 196 (11.3) | 2982 (14.2) | 0.90 (0.74, 1.09) | 0.26 | 96 (12.0) | 3882 (14.0) | 0.90 (0.69, 1.18) | 0.43 | 52 (12.3) | 4145 (13.9) | 0.86 (0.59, 1.26) | 0.44 |
| everyday | 742 (42.6) | 7318 (35.0) | 1.05 (0.92, 1.19) | 0.47 | 348 (43.6) | 10667 (38.4) | 1.00 (0.83, 1.20) | 1 | 176 (41.7) | 11646 (39.1) | 1.06 (0.82, 1.37) | 0.65 |
| Body mass index, n (%) |  |  |  |  |  |  |  |  |  |  |  |  |
| 18.5-22.9 | 473 (26.8) | 9675 (42.2) | 1.00 (reference) |  | 138 (17.1) | 11657 (39.0) | 1.00 (reference) |  | 208 (40.5) | 11840 (37.2) | 1.00 (reference) |  |
| <18.5 | 46 (2.6) | 1080 (4.7) | 0.81 (0.59, 1.13) | 0.22 | 16 (2.0) | 1264 (4.2) | 1.04 (0.60, 1.82) | 0.89 | 24 (4.7) | 1223 (3.8) | 0.83 (0.47, 1.46) | 0.51 |
| 23.0-27.5 | 935 (53.1) | 9710 (42.3) | 2.00 (1.76, 2.26) | <0.001 | 409 (50.7) | 13142 (43.9) | 2.57 (2.07, 3.18) | <0.001 | 213 (41.5) | 14238 (44.8) | 0.89 (0.70, 1.11) | 0.3 |
| >27.5 | 308 (17.5) | 2475 (10.8) | 2.80 (2.38, 3.29) | <0.001 | 244 (30.2) | 3864 (12.9) | 5.50 (4.37, 6.92) | <0.001 | 68 (13.3) | 4500 (14.2) | 0.88 (0.63, 1.22) | 0.45 |

Abbreviation: CI = Confident interval. HR = Hazard ratio.

* the HR and P values were calculated using Cox regression models adjusted by age, sex (male and female), rurality (urban and rural), education level (classified as primary school and below, middle school, college and above), marital status (married, single, divorced and widowed), cigarette smoking (non-smoker, current smoker, former smoker), alcohol consumption (non-drinker, occasionally drinker, frequently drinker, and daily drinker), exercise frequency (none, occasionally, frequently, everyday) and body mass index (normal weight: 18.5-22.9, underweight: <18.5, overweight: 23.0-27.5 and obese: >27.5 kg/m2).

**Fig.S1 Age pyramid of the Guangdong Biobank cohort**

The background population was derived from the officially registered residents of Xiaolan Town in 2016.


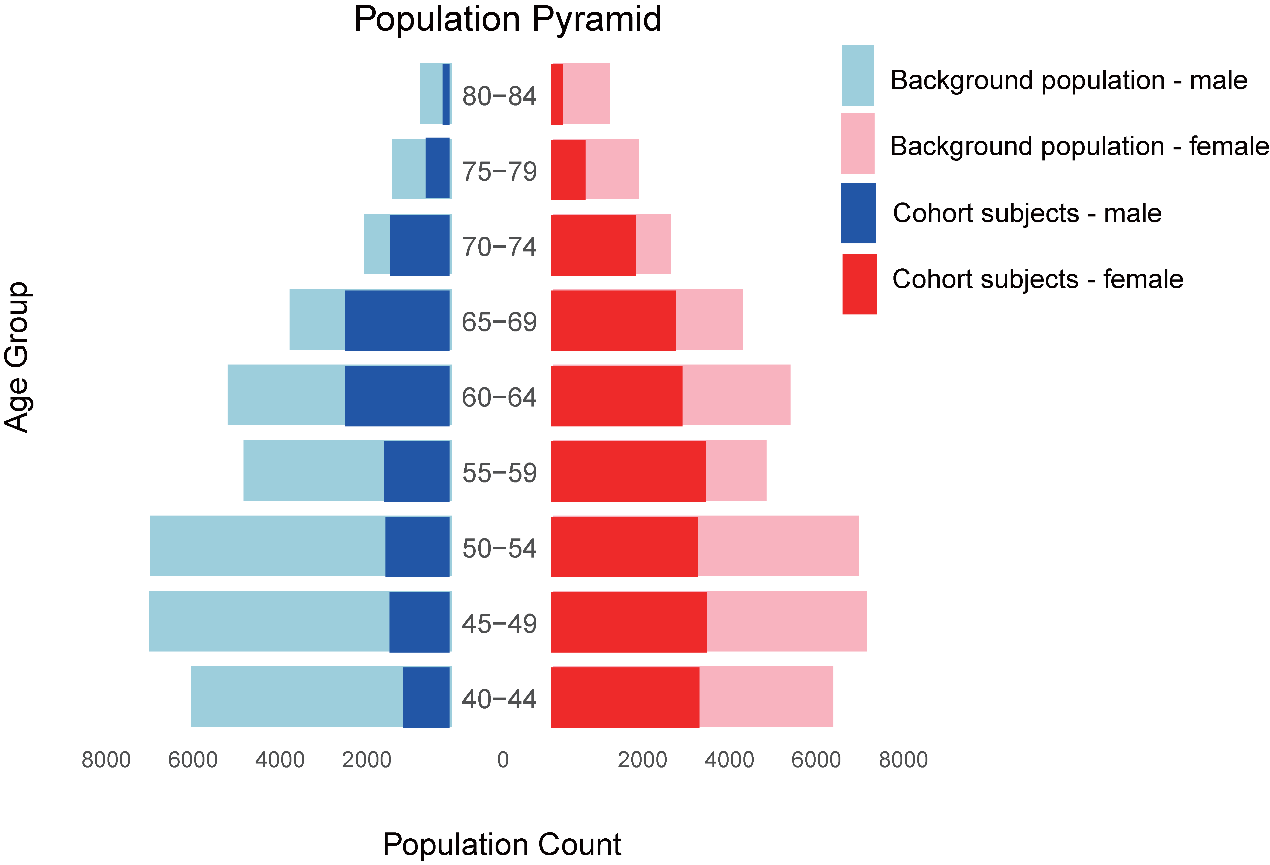


**Fig.S2 Principal components analysis of the genotyped sub-cohort in the Guangdong Biobank Cohort.** (a) Plot of the first and second principal component; (b) Plot of the second and the third principal component. (c) The fraction of variance explained by top 20 principal components. The principal components (PCs) were calculated using high-quality autosomal SNPs after linkage disequilibrium (LD) pruning. Each solid circle represents an individual.


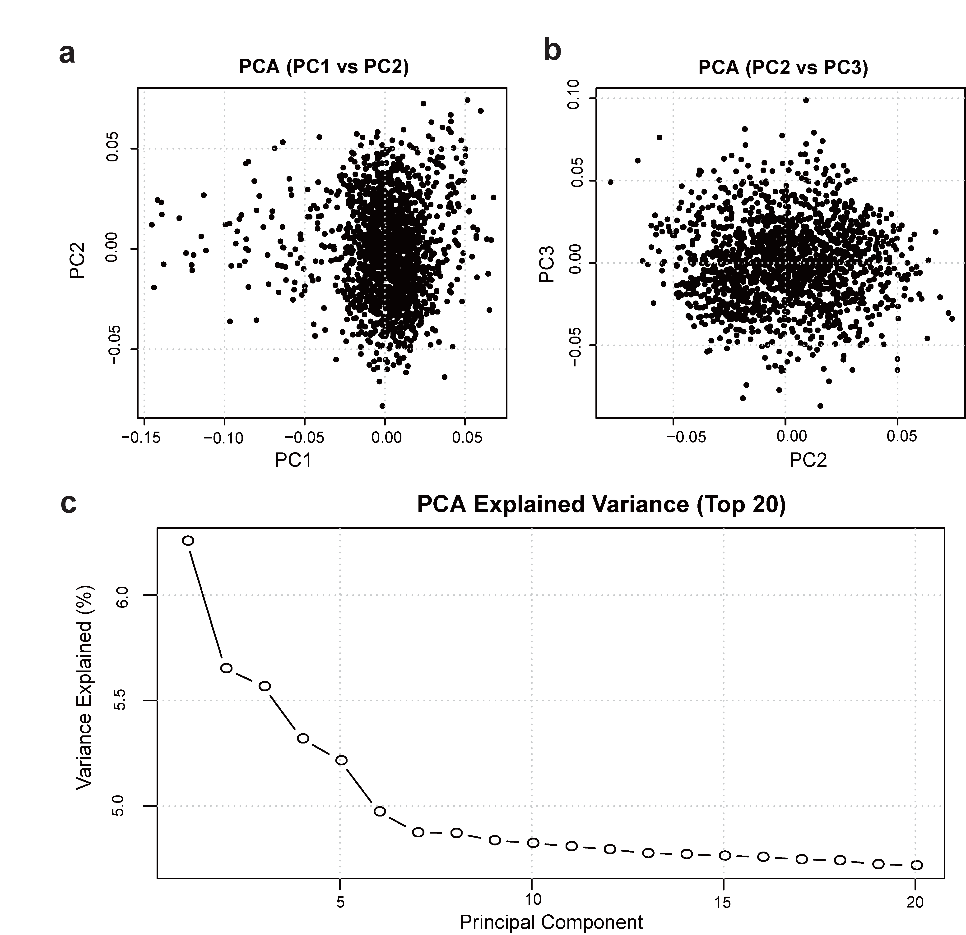


**Fig.S3 Bar plots for the distribution of the runs of homozygosity (ROH)** (a) Numbers of the ROH segments; (b) Length distribution of the ROH.


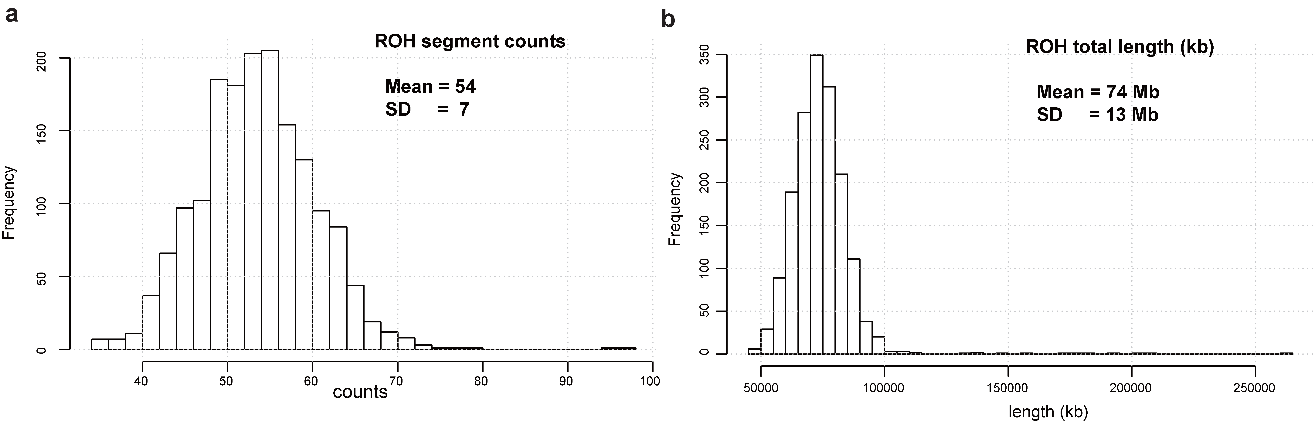


**Fig.S4 Quantile-Quantile (QQ) plots of (a) randomly simulated phenotype and (b) sex.**


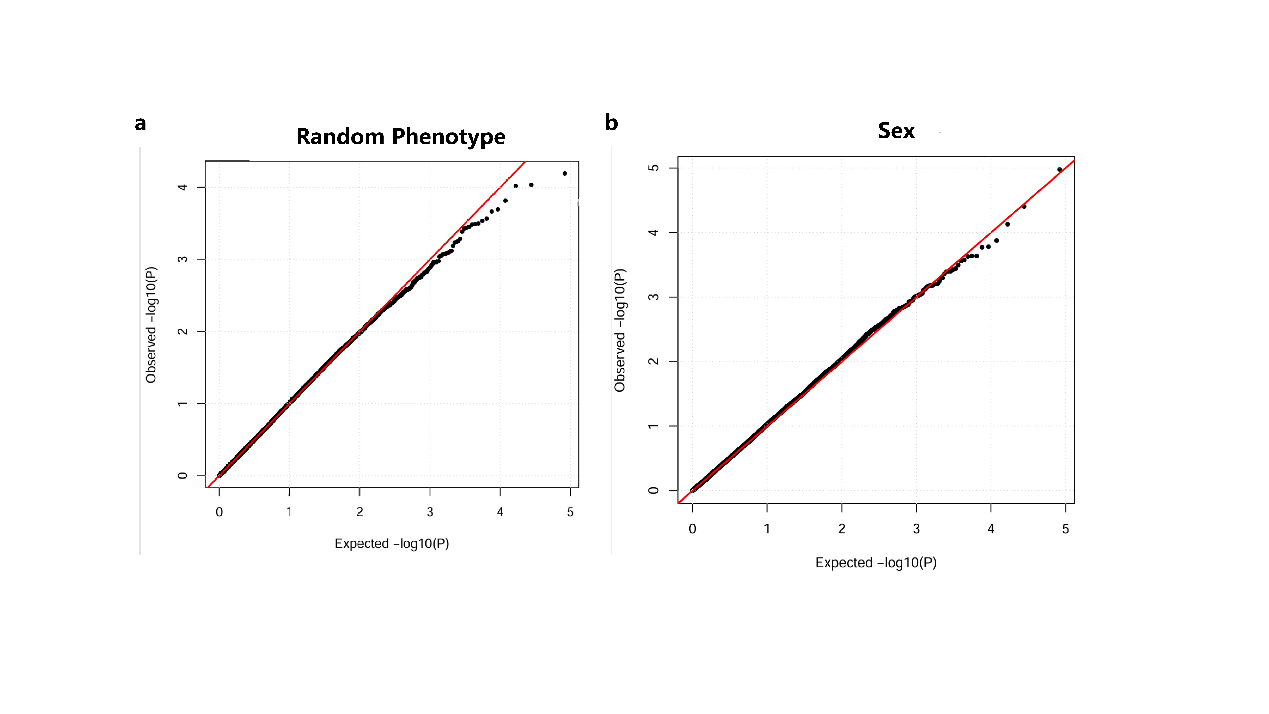

Supplement: Supplementary file 1 — Supplementary Material 1 [file 10654_2025_1320_MOESM1_ESM.docx]
